# The Utility of Body Composition Assessment in Nutrition and Clinical Practice: An Overview of Current Methodology

**DOI:** 10.3390/nu13082493

**Published:** 2021-07-22

**Authors:** Clifton J. Holmes, Susan B. Racette

**Affiliations:** 1 Program in Physical Therapy, Washington University School of Medicine, St. Louis, MO 63110, USA; racettes@wustl.edu; 2 Department of Medicine, Washington University School of Medicine, St. Louis, MO 63110, USA

**Keywords:** nutritional status, obesity, sarcopenia, osteoporosis, bioimpedance, anthropometrics

## Abstract

Body composition is a key component for maintaining good general health and longevity. It can be influenced by a variety of factors, including genetics, environment, and lifestyle choices. The assessment of body composition is an essential tool for nutrition specialists to effectively evaluate nutritional status and monitor progression during dietary interventions. As humans age, there is a natural increase in fat mass coupled with a gradual decline in lean mass, specifically in bone and muscle mass. Individuals with a high body fat percentage are at a greater risk of cardiovascular diseases, type 2 diabetes, several types of cancer, and early mortality. Significant decreases in bone mineral density signify osteopenia and osteoporosis, while reductions in skeletal muscle mass increase the risk of developing sarcopenia. Moreover, undernutrition exacerbates the effects of many medical conditions and is important to address. Though weight tracking and calculation of BMI are used commonly by clinicians and dietitians, these measures do not provide insight on the relative contributions of fat mass and fat-free mass or the changes in these compartments that may reflect disease risk. Therefore, it is important that healthcare professionals have a critical understanding of body composition assessment and the strengths and limitations of the methods available.

## 1. Introduction

An individual’s nutritional status is defined as “the condition of the body, resulting from the balance of intake, absorption, and utilization of nutrients and the influence of particular physiological and pathological status” [1,2]. The assessment of nutritional status has major relevance at both the individual level, as is the case for clinical practice, and among populations, as used in epidemiologic and public health research, to determine the presence of increased risk for nutrition-related conditions [2]. Anthropometric and body composition assessments are used to routinely detect or diagnose several important nutritional problems among adults and youth, including being overweight, obesity, undernutrition, osteoporosis, sarcopenia, and sarcopenic obesity.

When determining a patient or client’s nutritional status, nutrition specialists (e.g., dieticians, expert clinicians) begin with a nutritional risk screening. The tools used in this screening assessment must be easy to use, time efficient, accurate, and standardized, allowing for adequate reliability between different assessments and assessors without a substantial increase in measurement error. Because there is no “silver bullet” measurement tool, practitioners are prompted to use a holistic approach to increase the validity of the screenings. Clinicians and dieticians most commonly use recent weight loss, current body mass, recent food intake, and medical history questionnaires during daily routine assessments [3,4]. Additional nutritional risk screening tools exist, including the Nutritional Risk Screening 2002, the Malnutrition Universal Screening Tool, and the Mini Nutritional Assessment, each one varying in its intended population [3,5]. However, as useful as the aforementioned tools are, certain inherent weaknesses exist amongst them, such as subjectivity, lack of sensitivity and precision to subtle changes in nutritional status, and inability to account for specific tissues of the body (e.g., fat mass, bone mineral content, and skeletal muscle mass). For these reasons, clinicians, dieticians, and other nutrition specialists should look to incorporate specific body composition assessment techniques for further insight into an individual’s nutritional status.

Body composition assessment divides an individual’s total body mass into the relative proportions of fat mass (FM) and fat-free mass (FFM); FFM is comprises muscles, bones, organs, ligaments, tendons, and water. The quantification of fat, muscle, bone, and water are highly informative in the diagnosis, management, and treatment of several nutrition-related conditions that impact individual and population health. Once an individual’s nutritional status has been determined following a nutritional risk screening, clinicians, dieticians, and other practitioners are then responsible for developing a protocol to achieve certain health-related outcomes. These outcomes can range from weight loss in overweight or obese clients with or at-risk for diabetes mellitus type 2 to significant increases in lean tissue mass or bone mineral density in patients with sarcopenia or osteoporosis. Though baseline assessments allow for the initial construction of a dietary program, frequent adjustments and the tracking of changes are necessary throughout its duration in order to gauge progression and establish realistic short-term goals. Understanding the best body composition assessment techniques and practices will allow nutrition specialists to perform these duties more effectively. Therefore, the aim of this review is to provide an overview of various body composition assessment methods that are relevant to common nutrition-related conditions.

## 2. Clinical Relevance of Body Composition Assessment

The primary purpose of measuring body composition in clinical settings is to evaluate nutritional status through the quantification of FM, FFM, bone mineral content, and/or body water (intracellular and extracellular). Assessment of nutritional status is recommended in clinical practice for hospitalized patients and at-risk outpatients with nutrition-related conditions [5,6]. While there is currently not a universally accepted best method for the assessment of nutritional status [3,7,8], body composition provides valuable information that contributes to the identification, diagnosis, and management of several medical conditions for which nutrition therapy is indicated. A general understanding of the methodology, advantages, limitations, and impracticalities of current and emerging body composition assessment tools is beneficial for clinicians, dietitians, and other practitioners who play an integral role in nutrition management. Moreover, with healthcare becoming more outcome-driven, it is necessary that valid and reliable methods are used to evaluate the efficacy of various nutrition interventions.

Obesity is classified as a worldwide epidemic that adversely affects health and longevity. Excess adiposity increases the risk of cardiovascular diseases [9], type 2 diabetes [10], several types of cancer [11], and early mortality [12]. In the United States, more than 70% of adults are classified as overweight or obese based on body mass index (BMI) [13], with over $100 billion spent annually on medical costs related to obesity [14]. Obesity has even been associated with a higher risk of hospitalization and placement on mechanical ventilation among individuals with coronavirus disease 2019 (COVID-19) [15]. Body composition assessment enhances the diagnosis of obesity and the monitoring of responses to obesity treatment programs, which is highly relevant to the management of obesity-related chronic diseases.

Despite the high prevalence of obesity, undernutrition and malnutrition are significant nutritional problems that are often detected with anthropometric and/or body composition assessments. Chronic undernutrition is defined as a progressive decrease in both FFM and FM that diminishes the general quality of health [16]. This condition is more prevalent in low-resource settings and can be attributed to multiple factors, including food insecurity, poverty, and illness [17,18,19]. Undernutrition has been linked to negative impacts on morbidity, hospital stay duration, quality of life, health care costs, and mortality [16,20,21,22,23]. The prevalence of undernutrition among older adults, patients with various chronic diseases, and hospitalized patients has continued to increase over time, with at least one-third of admitted adult patients being malnourished [16,17,20,21,22,23]. According to a meta-analysis involving 240 studies, malnutrition has been reported in 3.0% of community dwelling older adults, 6.0% of those in outpatient care, 8.7% among those receiving home care services, 22.0% of those with in-hospital stay, 17.5% of those in nursing homes, and 29.4% in rehabilitation/sub-acute care [24].

In the U.S. and Europe, it has been reported that 30% of women have osteoporosis, and that 40% of post-menopausal women and 30% of men will experience osteoporotic fractures [25]. Osteoporosis is a major public health problem contributing to fractures, loss of independence, and early mortality. Generally, individuals with osteoporosis share the characteristics of being underweight through severely diminished lean tissue and fat mass stores [26]. In the past, clinicians used low BMI values in older men (<20.9 kg/m^2^) and women (20.1 kg/m^2^) to evaluate the risk of losing basic living skills and developing osteoporosis [26]. Over the years, because its diagnosis is based on the quantification of bone mineral density, dual-energy X-ray absorptiometry (DXA) has become the criterion indicator. The accuracy and precision of DXA scans can give clinicians and dieticians greater knowledge of an individual’s status. Classification as having normal bone mineral density, osteopenia, or osteoporosis allows for nutrition specialists to better prescribe dietary and supplemental intake changes of calcium, vitamin D, and other nutrients to treat the disease [27].

Another condition plaguing many older adults is sarcopenia, which is characterized by reduced FFM, with a muscle mass index that is more than two standard deviations below the sex-specific reference in a young, healthy population [2]. In addition to the observed significant atrophy of skeletal muscle, there are simultaneous decreases in muscular strength and overall physical function, further increasing the risk of injury, disability, and mortality [28]. Both sarcopenia and sarcopenic obesity, in which reduced FFM is combined with increased FM, are recognized as important nutritional problems that place older adults at higher risk of morbidity and mortality [2,29]. The combination of low muscle mass and excess adiposity in an older adult poses significant adverse consequences on functional capacity, resulting in diminished cardiorespiratory fitness and declining health status [30]. Importantly, because BMI does not distinguish FM from FFM, it often does not identify sarcopenia, particularly if the BMI value is in the healthy or overweight range. Having accurate assessments of body composition will allow practitioners to monitor lean tissue changes during a nutrition intervention and more efficiently prescribe protein intake patterns and physical activity to promote optimal muscle building. Moreover, in the case of sarcopenic obesity, simultaneous goals exist to increase skeletal muscle mass while also decreasing fat mass. Greater nuance is needed by clinicians and dieticians to ensure that significant muscle mass is not lost during weight loss phases and that a significant increase in body fat percentage does not occur during weight gain phases. Therefore, it is important to use a body composition assessment method that quantifies FM and FFM.

The routine assessment of body composition has also shown clinical significance for the treatment of cancer, specifically as it pertains to cachexia and lymphedema. Cancer cachexia falls under the umbrella of sarcopenia, where it is defined as a multifactorial syndrome that causes extreme weight loss and muscle wasting, with or without the loss of fat [31,32]. Cachexia can occur in up to 80% of patients with advanced stage cancer and has been associated with nearly 20% of cancer-related deaths [33]. For the screening and monitoring of cancer-induced cachexia, measuring total caloric and macronutrient amounts are recommended in conjunction with regular and consistent tracking of weight and BMI changes. In 2017, the European Society of Clinical Nutrition and Metabolism published evidence-based guidelines for nutritional care and recommended that body composition measures be added to expand nutrition-related assessments, aiding clinicians in treating patients with cancer cachexia and researchers investigating the condition [34]. With further progression, computer tomography (CT) is used for more in-depth analysis of body composition changes [35].

Lymphedema is another condition most commonly observed during the treatment of cancer [36]; however, increasing evidence has shown the potential link between lymphedema and obesity [37]. Lymphedema refers to the swelling present in the arms or legs caused by damage or the removal of a patient’s lymph nodes, resulting in a blockage in the lymphatic system that prevents lymph from draining properly [37]. Due to the swelling located in the limbs of patients with lymphedema, segmental bioelectrical impedance analysis (BIA) has demonstrated significant relationships with clinical measurements and can be used as a practical tool for monitoring individuals during treatment [36,38].

Treatment for all of these conditions—obesity, undernutrition, osteoporosis, sarcopenia, sarcopenic obesity, cachexia, and lymphedema—necessitates accurate diagnosis to implement the proper nutritional intervention and therapy. For these reasons, nutritional management of at-risk patients should integrate strategies to accurately and reliably assess body composition using a cost-effective medico-economic approach [6,39,40].

## 3. Methods for Estimating and Quantifying Body Composition

Several of the most commonly-used body composition assessment methods are presented in this review. Table 1 lists each method, the equipment needed, the assessment time required, and the pros and cons of each. Anthropometric methods that provide proxies for body composition, such as BMI and circumferences, are included for completeness, as they are widely available, widely used, and have reference values that signify different levels of health and disease risk.

Most body composition assessment methods are based on a two-compartment model that separates the body into fat and fat-free components (i.e., FM and FFM, respectively). Historically, hydrostatic weighing (HW) was the gold-standard assessment technique, but the equipment, space, expertise, and time required, combined with high participant burden, made HW impractical in most non-research settings. Dual-energy X-ray absorptiometry is currently the preferred criterion measure and has the advantage of quantifying bone mineral content in addition to FM and FFM, making this a three-compartment, or multi-compartment, model. Alternative methods for assessing body composition include skinfolds (SKF), BIA, digital image analysis, air displacement plethysmography (ADP), and sophisticated imaging techniques such as CT and magnetic resonance imaging (MRI). Attributes that guide the optimal choice of body composition assessment method include accuracy, reliability, the condition for which it is being used, accessibility, cost, ease of use, participant burden, and participant safety.

### 3.1. Body Mass Index (BMI)

Body mass index is the most commonly used anthropometric method to assess weight-related health risk. The BMI method classifies individuals into specific weight status categories that are associated with different levels of health risk (see Table 1) [41]. Advantages of BMI include the relative ease of performing height and weight measurements, low cost, minimal participant burden, and standardized classification of health risk based on large reference datasets. The utility of BMI is not only for classifying weight status, but also for tracking changes at the individual or population-level over time, particularly in response to individualized treatment or public health measures. The primary limitation of BMI is that it does not distinguish between FM and FFM [42] and therefore may misclassify some older adults as being a healthy weight (i.e., those with increased FM and decreased FFM) and some athletes as being overweight (i.e., those with increased muscle mass and relatively low FM).

### 3.2. Waist and Hip Circumferences

Circumference measures require only a tape measure to quantify the circumferences of various anatomical locations, with waist circumference being one of the most notable measures. Waist circumference can be used to identify excess abdominal adiposity, with values >80 and >88 cm in women and >94 and >102 cm in men reflecting increased risk and substantially increased risk, respectively, for metabolic complications [43,44]. Waist-to-hip ratio (WHR) and waist-to-height ratio (WHtR) also reflect excess abdominal adiposity when WHR values are ≥0.85 in women or ≥0.90 in men or when WHtR ≥0.50 (women and men). High values for these measures are associated with various chronic cardiovascular and metabolic diseases as well as early mortality [42]. Although circumference measures are informative and practical to obtain, they do not quantify FM and FFM and therefore do not provide a measure of body composition.

### 3.3. Skinfolds (SKF)

**Figure 1:**
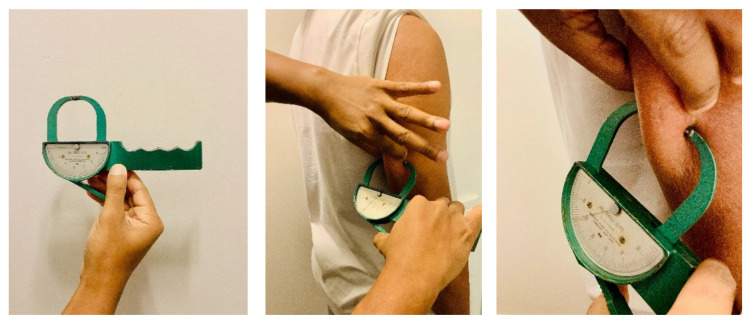
Skinfold measurement at triceps site; photo courtesy of Clifton J. Holmes, PhD.

The SKF technique is an inexpensive method to estimate %Fat by measuring the thickness of skinfolds at different sites of the body using calipers. This method is based on the principle that the amount of subcutaneous fat is proportional to the amount of total body fat. The specific skinfold sites and number of sites vary depending on which equation is used to estimate body density (Db). Common Db equations are sex-specific and include three, four, or seven skinfold sites; some also include circumference measurements. As examples, a three-site method for men may include triceps, chest, and subscapular skinfolds or chest, abdomen, and thigh skinfolds; three sites for women may include triceps, abdomen, and suprailiac skinfolds. A common seven-site method for men and women includes chest, midaxillary, triceps, subscapular, abdomen, suprailiac, and thigh skinfolds [45,46,47]. To perform this technique, the assessor pulls the fold of skin and subcutaneous fat away from the underlying muscle and then places a skinfold caliper over the fold. The pressure-sensitive caliper adjusts to the skinfold thickness and provides a measurement in mm. Metal calipers are preferred (see Figure 1); plastic calipers also are available. To compute %Fat, FM, and FFM, the SKF measurements are summed and inserted into the applicable sex-specific Db equation. The %Fat is then calculated from the Db using one of the following equations [48,49]:

%Fat = [4.95/Db − 4.50] × 100 or %Fat = [4.570/Db − 4.142] × 100

### 3.4. Bioelectrical Impedance Analysis (BIA)

**Figure 2:**
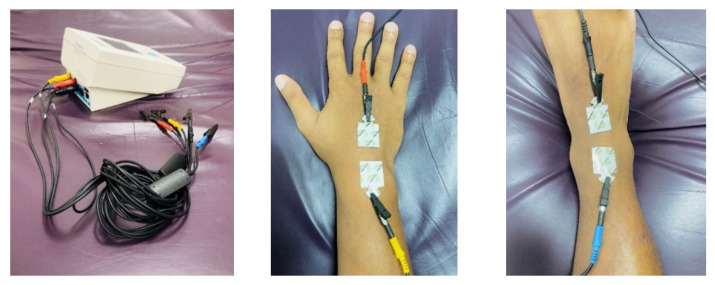
Laboratory-based bioimpedance device; photo courtesy of Clifton J. Holmes, PhD.

Bioelectrical impedance analysis involves a low-level electrical current that passes through an individual’s body while impedance, or opposition to the flow of the current, is measured. The electrical current flows readily through aqueous compartments because electrolytes in body water conduct this current; fat tissue causes resistance to the current flow. Lean tissue mass comprises approximately 73% water, whereas fat mass has extremely low hydration based on its chemical composition. Therefore, individuals with a high proportion of FFM (i.e., low %Fat) with proper hydration through intracellular and extracellular water have lower resistance and greater reactance measures than those with high %Fat. The sum of the resistance and reactance measured within an individual provides impedance values. Because BIA is extremely sensitive to total body water, measures should be consistently taken in similar states of hydration to reduce error (e.g., fasted, upon waking in the morning). BIA devices quantify impedance, from which they estimate intracellular water, extracellular water, total body water, FFM, FM, and %Fat. BIA devices vary in sophistication and features; some use single-frequency electrical currents, while others implement multi-frequency currents for greater penetration of different tissues and therefore with greater accuracy (see Figure 2) [50,51]. Some BIA instruments provide a measure of whole-body FM and FFM only, others provide segmental assessments of FM and FFM in each limb and the trunk, in addition to whole-body measures, and other BIA devices provide estimates of bone mineral content in addition to FM and FFM. Additionally, the raw BIA variable of phase angle, (i.e., ratio of resistance to reactance) provided with certain BIA devices, has gained significance for its potential application in both sports and healthcare. It can change based on the interface between cell membranes and tissues and be used as an index for water distribution, body cell mass, and cellular integrity. Phase angle has been associated with many nutritional markers and can provide viable insight to nutritional status and the effects of the supplementation strategies used by clinicians and dietitians [52].

**Figure 3:**
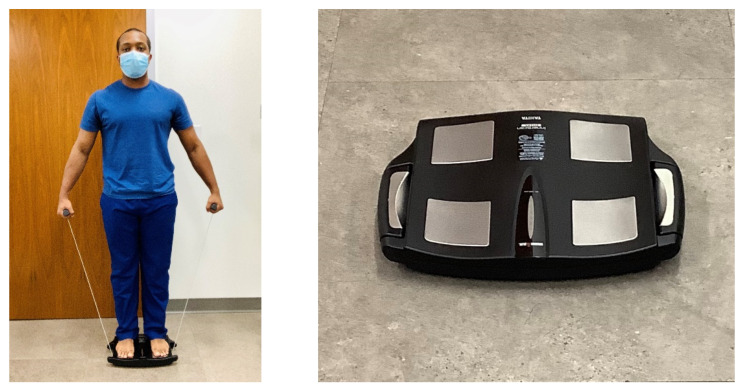
Home-based bioimpedance device; photo courtesy of Clifton J. Holmes, PhD.

Accuracy and reliability vary widely among BIA instruments. A number of studies have demonstrated the validity of both single-frequency and multi-frequency devices, concluding that BIA may be used as an alternative to DXA for whole-body and segmental body composition assessment in large groups (see Figure 3) [53,54]. However, single-frequency devices and segmental measures demonstrate the largest differences when compared to DXA, with the inaccuracy increasing in conjunction with higher levels of BMI [55,56,57,58]. Previous research has also shown BIA scales to give inaccurate estimates of bone mineral content in comparison to DXA [59,60], while other research has demonstrated that BIA-derived bone mineral content can be used in multi-compartment models in place of DXA [61]. The accuracy of the devices is also affected by the regression used by the device in question, with many manufacturers using their own equations derived during the internal validity testing of the product in question. However, many of these equations are general, with little specificity to varying populations. Finally, many BIA instruments are portable, while the more sophisticated models designed for clinical and research settings are less portable [62]. Although relatively inexpensive compared to DXA and ADP instruments, the cost among the wide variety of BIA devices varies significantly, depending on the features.

### 3.5. Digital Image Analysis

Three-dimensional (3D) body scanner devices and smartphone digital image analysis applications have emerged as relatively quick and easy-to-use techniques for body composition analysis. Whole-body optical scanners use digital imaging to estimate body volume, size, and circumferences at various anatomical locations to estimate body composition metrics [63]. Some 3D optical scanners have been validated against multi-compartment models, such as DXA, ADP, and BIA. However, because of the novelty of these devices and applications, more validation research is needed in a wide range of populations and during longitudinal tracking of body composition.

### 3.6. Air Displacement Plethysmography (ADP)

**Figure 4:**
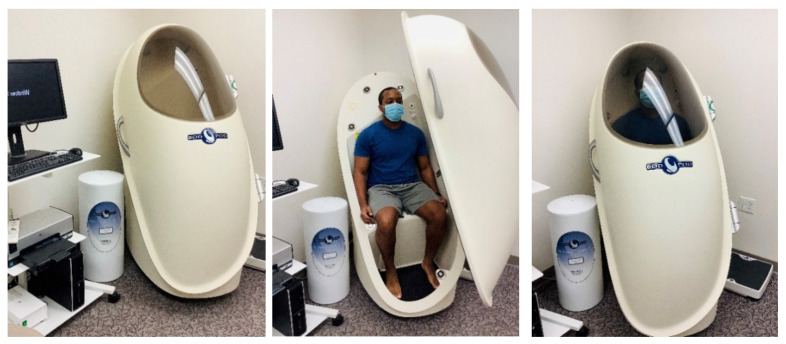
Air displacement plethysmography via BOD POD; photo courtesy of Clifton J. Holmes, PhD, in conjunction with the Pediatric Clinical Research Unit at the Washington University School of Medicine in St. Louis.

Air displacement plethysmography, performed using the BOD POD, uses densitometry to estimate body composition [64] (similar to SKF and HW). The BOD POD contains a chamber with a volume of 450 or 500L and computerized sensors that measure body volume by air displacement while the individual is seated in the chamber (see Figure 4). Thoracic gas volume (i.e., the volume of air in the lungs and thorax) can be quantified as an additional measure while in the chamber or estimated by the BOD POD. Once all of the necessary variables are measured, the BOD POD program calculates ~8 body composition metrics. The accuracy of the BOD POD has been deemed high, but it has been shown to overestimate %Fat in lean individuals in some studies when compared to HW [65,66,67]. Potential contributors to inconsistent measures include testing conditions, clothing worn in the BOD POD, and excessive facial or body hair [50,51].

### 3.7. Dual-Energy X-ray Absorptiometry (DXA)

**Figure 5:**
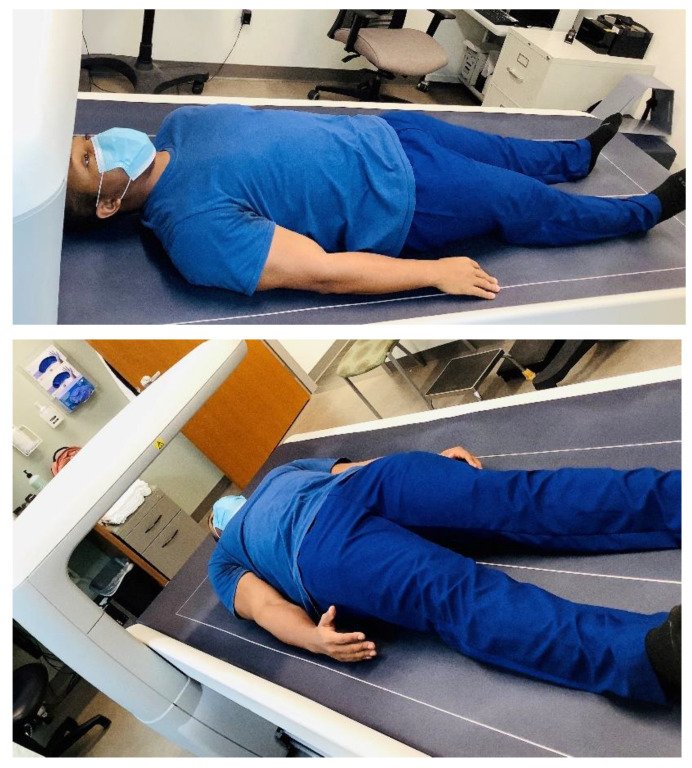
Dual-energy X-ray absorptiometry; photo courtesy of Clifton J. Holmes, PhD, in conjunction with the Clinical Translational Research Unit at the Washington University School of Medicine in St. Louis.

DXA has become the preferred method for assessing body composition, with a notable advantage being that it provides a multi-compartment assessment that includes bone [68,69]. Multi-compartment models have the ability to distinguish multiple tissue components, which reduces the assumptions from which body composition estimates are based and increases the accuracy [70,71]. The DXA instrument measures tissue absorption of high- and low-energy X-ray beams that pass through the individual lying supine on the scanning bed (see Figure 5). It uses the attenuation, or weakening, of those X-ray beams to provide accurate estimates of bone mineral content and soft-tissue composition. This attenuation of the X-ray beams is measurable and heavily dependent on the thickness, density, and chemical composition of the underlying tissue. The major limitation of DXA is radiation exposure (albeit a very low dose). With DXA, practitioners can quantify FM, bone mineral content, and non-bone, fat-free tissue mass, thereby reducing the assumptions being relied on with SKF, ADP, and HW methods [68]. However, DXA is unable to assess total body water and therefore assumes a constant FEM hydration level and measures excess body water as additionally lean tissue mass, indicative of a source of error.

### 3.8. Computed Tomography (CT) and Magnetic Resonance Imaging (MRI)

**Figure 6:**
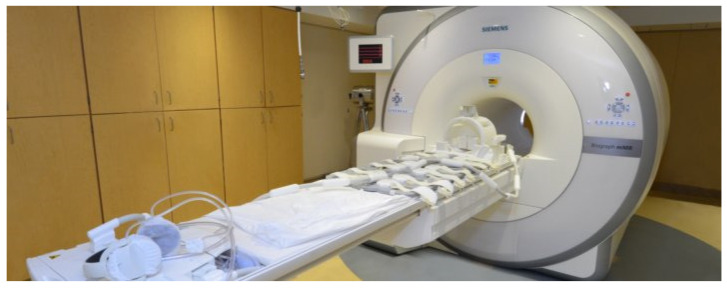
Positron Emission Tomography and Magnetic Resonance Imaging scanner; photo courtesy of the Center for Clinical Imaging Research in the Mallinckrodt Institute of Radiology at Washington University School of Medicine in St. Louis.

CT and MRI are imaging techniques that provide cross-sectional images of specific body regions in the form of a plane through the body (see Figure 6) [51,72]. The results from these imaging methods are considered the most accurate means of quantifying body composition at the tissue–organ level and have significantly impacted the scientific understanding of body composition and its relation to disease risk and outcome [51,73]. Both CT and MRI allow for the segmentation of specific tissues and provide direct measures of a tissue cross-sectional area [72]. The CT system comprises an X-ray tube and receiver, both of which rotate in a perpendicular plane to the patient. Similar to the DXA scan, X-rays are emitted from the tube and are attenuated as they flow through the targeted tissues [51,74]. Once the receiver identifies the X-rays, image reconstruction commences using various mathematical techniques. Pixelated cross-sectional images of the target area are illustrated in gray scale, which reflects the composition of the tissue and differs based on the density of the tissues. Similar to a DXA scan, a CT scan exposes the patient to a relatively low-dose of radiation, though it is greater than that of DXA. For example, the average effective dose for a DXA bone density test is 0.001 mSv, while a CT scan of the spine or pelvis is 6 mSv. This higher exposure dosage makes the frequency of CT scans a limiting factor. While the CT system uses ionizing radiation, MRI relies on the interaction between protons and the magnetic fields produced by the MRI system’s instrumentation [51,72,74]. MRI uses the radio frequency signals resulting from the interaction between the protons of the tissues and the magnetic fields to generate cross-sectional images. Both CT and MRI provide valid and in-depth body composition information, but these imaging techniques require expensive scanners, certified, trained technicians to perform the scans, analysis software, and expertise to quantify the tissue–organ level components of the produced images. It is worth noting that one drawback of the MRI scan is the adherence of patients in remaining motionless for long periods of time (e.g., 20–120 min) in a small space with potentially loud noises caused by the working equipment. These obstacles presented by the MRI system can diminish compliance and make patients uncomfortable during testing.

### 3.9. Hydrostatic Weighing (HW)

**Figure 7:**
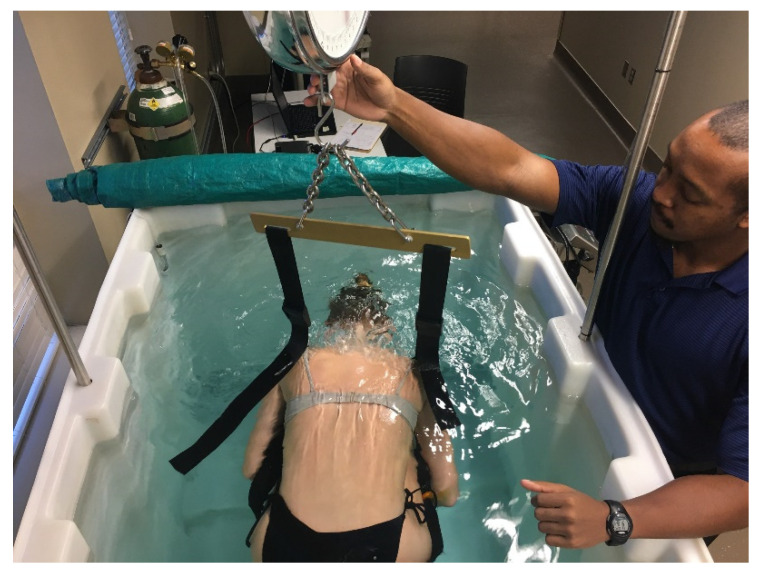
Hydrostatic weighing; photo courtesy of Clifton J. Holmes, PhD, in conjunction with the Exercise Physiology Laboratory in the Department of Kinesiology at the University of Alabama, Tuscaloosa.

Hydrostatic weighing (aka underwater weighing, hydrodensitometry) is a foundational body composition method that was considered the “gold standard” for assessing FM and FFM for decades. HW is based on Archimedes’ principle, which states that when a body is immersed in water, it is buoyed by a counterforce equal to the weight of the displaced water (see Figure 7) [50,51]. Basically, the weight of a person (or an object) in water is less than their mass on land. This difference in weight provides an estimate of body volume. Muscle and bone are denser than water, while fat tissue is less dense; therefore, the more the FFM and less FM someone has, the greater their underwater weight will be, and vice versa. Once the mass and volume of an individual are known, body density can be calculated as mass ÷ volume, and %Fat can be calculated using one of the body composition equations shown above for the SKF method [48,49]:

One additional, important component needed for HW assessment is residual lung volume, which is the amount of air remaining in the lungs after a maximal expiration (~1–2 L). Whether practitioners measure residual lung volume or estimate it using various prediction equations, this value must be included in the hydrostatic weighing calculations of body composition to avoid significant overestimations of %Fat. Because HW requires individuals to submerge themselves underwater repeatedly after expiring as much air from their lungs as possible, many find it to be extremely uncomfortable.

## 4. Practical Applications

Important factors to consider when choosing a body composition assessment method are accuracy, accessibility, and expense. Table 2 provides an overview of the various methods discussed above and provides each method’s requirements as well as the associated pros and cons. In order to achieve the greatest accuracy and reliability, it is important to choose the method that is the most appropriate for the tissue or condition of interest. For example, DXA is optimal for quantifying bone mineral density for the diagnosis and management of osteoporosis, whereas BIA, ADP, or DXA may be used to quantify FFM and FM for identifying sarcopenia, cachexia, or sarcopenic obesity. Moreover, BIA stands as an ideal tool for monitoring significant changes in hydration status and body fluid distribution among patients participating in a weight loss intervention and those with or at risk of developing lymphedema. With older adults comprising an increasing proportion of the population, the identification of these age-related conditions is particularly important. Any of the anthropometric and body composition assessment methods can be used to identify obesity, the most prevalent nutrition-related condition in the U.S. and one for which treatment and management approaches are implemented frequently. Likewise, this array of assessment methods can be used to track treatment responses during weight loss interventions for obesity. Waist circumference, alone or expressed as WHR or WHtR, is an effective and simple approach for identifying the excess abdominal adiposity that contributes to metabolic conditions such as type 2 diabetes, polycystic ovary syndrome, and non-alcoholic fatty liver disease. An MRI facilitates the quantification of intra-abdominal and subcutaneous adipose tissues, which pose distinct metabolic risks. SKF, BIA, and ADP are excellent options for quantifying the FFM and FM of athletes and for guiding nutritional strategies to optimize performance. A combination of anthropometric and more sophisticated assessment methods may be advantageous, particularly when tracking changes longitudinally in response to a diet or exercise program.

The accuracy of body composition assessment is optimized if the individual follows specific pre-testing guidelines: (a) no food within 8 h of testing, (b) no water within 2 h of testing, (c) no exercise within 24 h of testing, (d) no alcohol consumption within 48 h of testing, and (e) empty the bladder/bowels within 30 min before testing. It is recommended to perform body composition assessments in the morning after an overnight fast, which helps to ensure that these conditions are met. Additionally, adequate (but not excessive) hydration is important for accurate assessment and can be estimated by checking the color of urine; if the urine color is dark, the individual should drink water and wait 30–45 min before being assessed (when feasible) [50,51]. Although accuracy is arguably the most important factor when choosing a body composition method, accessibility and expense are important considerations.

## 5. Conclusions

All of the body composition assessment techniques discussed in this article have some level of scientific literature validating them. Nevertheless, each of the aforementioned methods has limitations and all methods provide estimates of body composition that are dependent on a range of assumptions. Finally, it is worth noting that quantifiable and clinically meaningful changes in body composition take time to occur; therefore, the frequency of assessments should be determined based on the individual, the intervention, and the goals to be achieved. In summary, body composition assessment is an important tool for the identification of common nutrition-related conditions that impact individual and public health and provides valuable information about responses to treatment.

## Figures and Tables

**Table 1:** Body mass index and waist circumference classifications.

Weight Status Classification	Body Mass Index (kg/m^2^)	
Underweight	<18.5	
Severe Thinness	<16.0	
Moderate Thinness	16.0 to 16.9	
Mild Thinness	17.0 to 18.4	
Normal weight	18.5 to 24.9	
Overweight	25.0 to 29.9	
Obese, Class I	30.0 to 34.9	
Obese, Class II	35.0 to 39.9	
Obese, Class III	≥40.0	
Waist Classifications	Waist Circumference	
Risk Classification	Women	Men
Normal	<32 in. (80 cm)	<37 in. (94 cm)
Increased	≥32 in. (80 cm)	≥37 in. (94 cm)
Substantially increased	≥35 in. (88 cm)	≥40 in. (102 cm)

**Source:** Adapted from World Health Organization. 2008. Waist Circumference and Waist-to-Hip Ratio. Report of a WHO Expert Consultation. Geneva: WHO.

**Table 2:** Overview of Body Composition Assessment Technique Requirements, Pros, and Cons.

Assessment Method	Equipment Needed	Time Needed for Assessment	Pros	Cons
Body Mass Index (BMI)	stadiometer scale	≤3 min	quick simple inexpensive	does not differentiate between FM and FFM
Circumferences	flexible tape measure	≤5 min	quick simple inexpensive portable	does not differentiate between FM and FFM
Skinfolds (SKF)	skinfold calipers	10–20 min	accurate when performed by a skilled assessor inexpensive portable	technical expertise required to minimize intra- and inter-observer variability close proximity & skin contact required with various body regions accuracy is compromised by dehydration, edema, & muscle wasting
Bioelectrical Impedance Analysis (BIA)	BIA instrument stadiometer scale & electrodes *(for some BIA instruments)*	≤5 min	quantifies reginal body composition *(some models)* provides an estimate of body water simple for assessor & individual being assessed accessible at home *(some models)* relatively inexpensive *(prices vary)*	accuracy varies across instruments contraindicated in individuals with implantable electronic devices due to potential interference
Digital Image Analysis	3D scanner ~ or ~ smartphone with camera & digital image app	1–2 min	quick simple portable	limited validation research tight-fitting clothing must be worn
Air Displacement Plethysmography (ADP)	BOD POD instrument stadiometer scale	≤10 min	automated; minimal technical expertise needed minimal effort needed from participant	expensive large space required minimal and tight-fitting clothing and swim cap must be worn excessive facial or body hair may introduce error
Dual-Energy X-ray Absorptiometry (DXA)	DXA machine	10–30 min	quantifies bone mineral content & bone mineral density, in addition to FM & non-bone lean mass high reliability minimal effort needed from participant	expensive personnel training & certification required low-level radiation exposure contraindicated during pregnancy
Computed Tomography (CT)	CT scanner analysis software	variable *(depends on regions being scanned)*	quantifies tissue cross-sectional area high validity minimal effort needed from participant	expensive equipment personnel training & certification required radiation exposure contraindicated during pregnancy
Magnetic Resonance Imaging (MRI)	MRI scanner analysis software	variable *(depends on regions being scanned)*	quantifies abdominal FM and other regions of interest high validity	expensive equipment personnel training & certification required
Hydrostatic Weighing (HW)	large tank or pool filled with water chair suspended from a scale above the tank spirometer metabolic cart or nitrogen washout system *(for residual lung volume)* scale *(for body weight on land)* nose clips	30–45 min	accurate validated	expensive, sophisticated equipment, set-up, & maintenance required technical expertise required uncomfortable for participant not feasible for individuals who fear being underwater

## Data Availability

No new data were created or analyzed in this study. Data sharing is not applicable to this article.
